# Pharmacokinetic Modeling of Paracetamol Uptake and Clearance in Zebrafish Larvae: Expanding the Allometric Scale in Vertebrates with Five Orders of Magnitude

**DOI:** 10.1089/zeb.2016.1313

**Published:** 2016-12-01

**Authors:** Vasudev Kantae, Elke H.J. Krekels, Anita Ordas, Oskar González, Rob C. van Wijk, Amy C. Harms, Peter I. Racz, Piet H. van der Graaf, Herman P. Spaink, Thomas Hankemeier

**Affiliations:** ^1^ Division of Analytical Biosciences, Systems Pharmacology Cluster, Leiden Academic Centre for Drug Research, Leiden University, Leiden, The Netherlands.; ^2^ Division of Pharmacology, Systems Pharmacology Cluster, Leiden Academic Centre for Drug Research, Leiden University, Leiden, The Netherlands.; ^3^ IBL, Institute of Biology, Leiden University, Leiden, the Netherlands.; ^4^ Science and Technology Faculty, Analytical Chemistry Department, University of the Basque Country/EHU, Bilbao, Spain.; ^5^ ZF-screens B.V., Leiden, The Netherlands.

**Keywords:** zebrafish, paracetamol, pharmacokinetics, allometry, metabolism, drug discovery

## Abstract

Zebrafish larvae (*Danio rerio*) are increasingly used to translate findings regarding drug efficacy and safety from *in vitro*-based assays to vertebrate species, including humans. However, the limited understanding of drug exposure in this species hampers its implementation in translational research. Using paracetamol as a paradigm compound, we present a novel method to characterize pharmacokinetic processes in zebrafish larvae, by combining sensitive bioanalytical methods and nonlinear mixed effects modeling. The developed method allowed quantification of paracetamol and its two major metabolites, paracetamol-sulfate and paracetamol-glucuronide in pooled samples of five lysed zebrafish larvae of 3 days post-fertilization. Paracetamol drug uptake was quantified to be 0.289 pmole/min and paracetamol clearance was quantified to be 1.7% of the total value of the larvae. With an average volume determined to be 0.290 μL, this yields an absolute clearance of 2.96 × 10^7^ L/h, which scales reasonably well with clearance rates in higher vertebrates. The developed methodology will improve the success rate of drug screens in zebrafish larvae and the translation potential of findings, by allowing the establishment of accurate exposure profiles and thereby also the establishment of concentration–effect relationships.

## Introduction

Zebrafish and zebrafish larvae (*Danio rerio*) are increasingly used as a complementary vertebrate model at various stages of drug discovery and development, ranging from disease modeling and target validation, to drug safety, toxicology, and efficacy screenings.^1–6^ This is due to (1) their similarity to humans in terms of morphological, molecular, genetic, and pathological features; (2) their small size; (3) their easy and inexpensive maintenance; (4) the availability of a large set of genetic tools; (5) the limited ethical issues involved; and (6) the rapid development of optically transparent larvae, allowing high-throughput imaging-based phenotypic screening.

Although the zebrafish larva is a well-established vertebrate model for toxicity and safety purposes, its utilization has been limited due to the gaps in our understanding of basic pharmacokinetic (PK) processes like absorption, distribution, and clearance, that drive the exposure to drugs and drug metabolites.^1,3^ Although, expression patterns of enzymes that drive drug metabolism have been characterized in zebrafish,^7–10^ to date metabolism and excretion of drug or drug metabolites have never been quantified in zebrafish larvae.

The aim of this study was to develop a new methodology to characterize drug elimination processes in 3-day-old zebrafish larvae, using paracetamol, also known as acetaminophen, as a paradigm compound. A highly sensitive liquid chromatography-mass spectrometry (LC-MS/MS)-based method was developed to accurately measure concentrations of paracetamol and its metabolites in zebrafish larvae. The obtained data were subsequently analyzed with nonlinear mixed effects modeling techniques.

## Materials and Methods

### Study design

To characterize the PK of paracetamol in zebrafish larvae, two experiments were performed. At day 3 postfertilization (dpf), hatched larvae were transferred to 24-well plates, with each well containing five larvae in 2 mL of embryo medium (pH = 7.9) with 1 mM paracetamol. In experiment 1, 150 zebrafish larvae were used to determine the absorption of paracetamol by continuous drug exposure and in experiment 2, 60 zebrafish larvae were used to study the elimination of paracetamol after the larvae were transferred from drug-containing to drug-free medium. All measurements in the experiments were carried out in triplicates, and the measured drug and drug metabolite amounts are expressed per larva, but represent mean values obtained from five larvae simultaneously.

Zebrafish larvae were handled in compliance with the local animal welfare regulations and maintained according to standard protocols (zfin.org). Embryos were collected from family crosses of AB/TL wild-type strain and grown at 28°C in embryo medium (60 g/mL Instant Ocean sea salts; Sera Marin) in the dark.

### Experiment 1

Zebrafish larvae were exposed to the drug-containing medium for 10, 20, 30, 40, 50, 60, 80, 100, 120, or 180 min. At the time of the measurement three replicates of five larvae were collected, and washed twice with 80/20 (v/v) water/methanol solution, after which excessive medium was removed. The samples were subsequently prepared for analysis as described below. Additionally, samples from the incubation medium were collected to study the excreted amounts of the metabolites. Larvae in control groups were exposed to the drug solution for a few seconds and to determine drug adherence to the skin.^11^

### Experiment 2

Larvae were exposed to the drug-containing medium for 1 h, washed with fresh medium to remove the residual drug solution, and then transferred to a drug-free medium. After 0, 1, 2, and 3 h of incubation, three replicates of five larvae were collected and washed twice in 80/20 (v/v) water/methanol solution after which excessive medium was removed and the samples were subsequently prepared for analysis as described below. Samples from the incubation medium were also collected at each time point.

This experiment was repeated a second time with three washing steps before transfer to drug-free medium, as the first time unchanged drug and metabolites appeared to have been transferred to the fresh medium. The second time, the duration of this experiment was also extended with sample collection up to 4 h after transfer to the drug-free medium. Data on paracetamol, paracetamol-sulfate, and paracetamol-glucuronide concentrations in the incubation medium from the first time the experiment was performed were excluded from the analysis as contamination was suspected.

### Sample preparation

The five zebrafish larvae of 3 dpf from a single well were added to 100 μL lysis buffer containing 90/10 (v/v) methanol/water solution with 45 ng/mL paracetamol-D_4_ internal standard (Sigma-Aldrich Chemie B.V., Zwijndrecht, Netherlands). Lysis was performed by snap-freezing each sample in liquid nitrogen followed by 2–3 min sonication. This procedure was repeated until all larvae were lysed and the sample was homogenous. The lysed samples were centrifuged for 10 min at 16,000 *g* at 4°C. Ninety microliter of supernatant was collected in a microcentrifuge tube, after which 72 μL water was added, yielding a sample of 50/50 methanol/water solution, which was injected to the LC-MS/MS system directly.

The medium samples (2 mL) from each well were collected in new eppendorf tubes and concentrated by speedvac. After reconstitution in 150 μL of 50/50 methanol/water solution, samples were injected to the LC-MS/MS system directly.

### Calibration curve

Paracetamol, paracetamol-sulfate, and paracetamol-glucuronide were purchased from Sigma-Aldrich Chemie B.V. Individual stock solutions at 1 mg/mL were prepared using methanol. Standard working solutions of 1000 ng/mL were prepared for these three compounds and for the paracetamol-D_4_ internal standard. On the day of analysis, the standard working solutions were further diluted to five additional calibration points and mixed with the internal standard solution to final concentrations of 9, 18, 45, 90, 180, and 270 ng/mL for paracetamol and its metabolites and 45 ng/mL for the internal standard. All calibration points were prepared in a composition similar to the lysis buffer (90/10 methanol/water). For constructing a matrix-matched calibration curve, 100 μL of each calibration point was spiked into a sample of five zebrafish larvae of 3 dpf and followed similar extraction procedure as described for the sample preparation above.

### Quantification of paracetamol and metabolites

Analyses were performed on an ultra performance liquid chromatography (UPLC) system (Acquity; Waters, Milford, MA) coupled to a quadrupole-time of flight (QTOF) mass spectrometer (MS; Synapt G2S; Waters, Wilmslow, United Kingdom) with an electrospray ionization (ESI) source. Positive ionization mode was used for the analysis of paracetamol and negative mode for the analysis of the paracetamol-sulfate and paracetamol-glucuronide metabolites.

Chromatography was performed at 40°C on a Waters Acquity UPLC HSS T3 column (1.8 μm, 2.1 × 50 mm) using as aqueous mobile phase a 0.01% formic acid solution (A) and acetonitrile (B) as organic modifier at a flow rate of 0.4 mL/min. The run time was 5 min, with the gradient at 100% A for the first 0.3 min, then B was linearly increased to 100% for the next 3 min and held for 0.5 min and finally the system was equilibrated to the initial conditions for 1.2 min. Injection volume was 5 μL and autosampler temperature was set to 10°C.

The MS method operated in both positive and negative polarities with separate injections in each polarity includes an MS scan function with a scan time of 0.2 s operated at a trap cell collision energy of 4 eV and a transfer cell collision energy of 2 eV. ESI voltage was set at 0.9 kV both for positive and negative modes. Cone voltage was set at 30 V and source offset at 80 V. The source temperature was set at 150°C with desolvation temperature at 500°C. Nitrogen was used for the cone, desolvation and nebuliser gases with settings of 50, 1000 L/h and 6 bar, respectively. Data were collected in continuum mode with a scan range of 50–850 m/z using the QTOF detector in high-resolution mode (∼20,000 full width at half maximum). About 0.1 mg/L Leucine enkephalin solution (acetonitrile: water 50:50 with 0.1% v/v formic acid) infused at a constant flow of 10 μL/min was used as the lock mass, a single point scan was collected every 10 s and averaged over three scans to perform mass correction (556.2771 m/z). The instrument was calibrated for both positive and negative polarities before analysis using sodium formate.

Masslynx 4.1 SCN916 (Waters) was used to acquire all the data and Targetlynx application to perform the quantitative analysis. The quantification of paracetamol and its metabolites was achieved by correcting the peak area response of paracetamol and its metabolites by the peak area response of the internal standard. The quantified values are expressed in terms of amount/larva. The developed method was sensitive with limits of quantification as low as 0.02 pmole/larva for paracetamol, 0.008 pmole/larva for paracetamol-sulfate, and 0.05 pmole/larva for paracetamol-glucuronide.

### Nonlinear mixed effects modeling

The nonlinear mixed effects modeling of paracetamol was performed using NONMEM 7.3 software (ICON Development Solutions, Ellicott City, MD),^12^ facilitated with Pirana^13^ and PsN.^14,15^ The First Order Conditional Estimation method was used in NONMEM. R 3.0.0 was used for graphical analysis and model diagnostics.

In a pooled analysis of the paracetamol concentrations in the larvae, a 0-order absorption rate was estimated to quantify the uptake of paracetamol from the incubation medium. A first-order clearance rate was estimated to quantify the elimination of paracetamol from the larvae. Both one and two-compartment models were tested to describe the distribution of paracetamol within the larvae. For both distribution models, the total distribution volume was fixed to 1, which meant for the two-compartment model that both compartments were expressed as fraction of the total volume of one larva. To quantify the residual variability an additive and a proportional error were tested, and a combination of both. Due to the destructive nature of the measurements, inter-individual variability in the PK processes could not be quantified.

The likelihood ratio test, based on the objective function value (OFV) in NONMEM was used for model selection. Assuming a χ^2^-distribution, a decrease in OFV of more than 3.84 points, corresponding to a significance level of *p* < 0.05 with one degree of freedom, was considered to be statistically significant. Precision of parameter estimates was evaluated based on standard errors obtained from the NONMEM output tables. Furthermore, the model fit was assessed by visual inspection of goodness-of-fit plots, these included plots of predicted versus observed paracetamol amounts in the zebrafish larvae, and of conditional weighted residuals^16^ versus time after start of the experiment and conditional weighted residuals versus predicted paracetamol amounts.

### Volume of zebrafish larvae

To enable the expression of paracetamol clearance in zebrafish larvae in absolute rather than relative terms, the average volume of the larvae was determined. For this, samples of 10, 20, 30, 40, 50, and 100 larvae at 3 dpf were placed onto parafilm M(R) (Bemis NA) with a transfer pipette after which the excessive water was removed with variable volume pipettes and filter paper. Subsequently, the weight of each sample was measured with a Mettler AE240 analytical balance. The average wet weight of the zebrafish larvae was determined using linear regression. To derive the average volume of the larvae, their density was assumed to be equal to the density of water at 25°C (0.997 g/mL).

### Comparing paracetamol clearance in vertebrate species

A search was performed in PUBMED using “paracetamol” OR “acetaminophen” AND “clearance”. Total paracetamol clearance values (CL) reported over the past 10 years in various vertebrate species were collected together with mean bodyweights (BWs) of the individuals included in a study. When detailed information on BW was not provided, average BW values from the species were obtained from literature. Data from disease models, combined drug formulation, or obese individuals were excluded. Distinctions were made between data collected in mature or immature individuals of a species.

All obtained CL were plotted versus BW on a double log scale. A linear model was fitted through the logarithm of the CL versus the logarithm of the BW in R (version 3.3.1) to obtain the parameters in the allometric equation below that describes the relationship between BW and paracetamol clearance [Eq. (1)].

\begin{align*}
CL = a \cdot B{W^{exp }} \tag{1}
\end{align*}

The fit was based only on CL from the higher vertebrates, excluding the zebrafish larvae.

## Results

### Quantification of paracetamol and metabolites

**FIG. 1.**
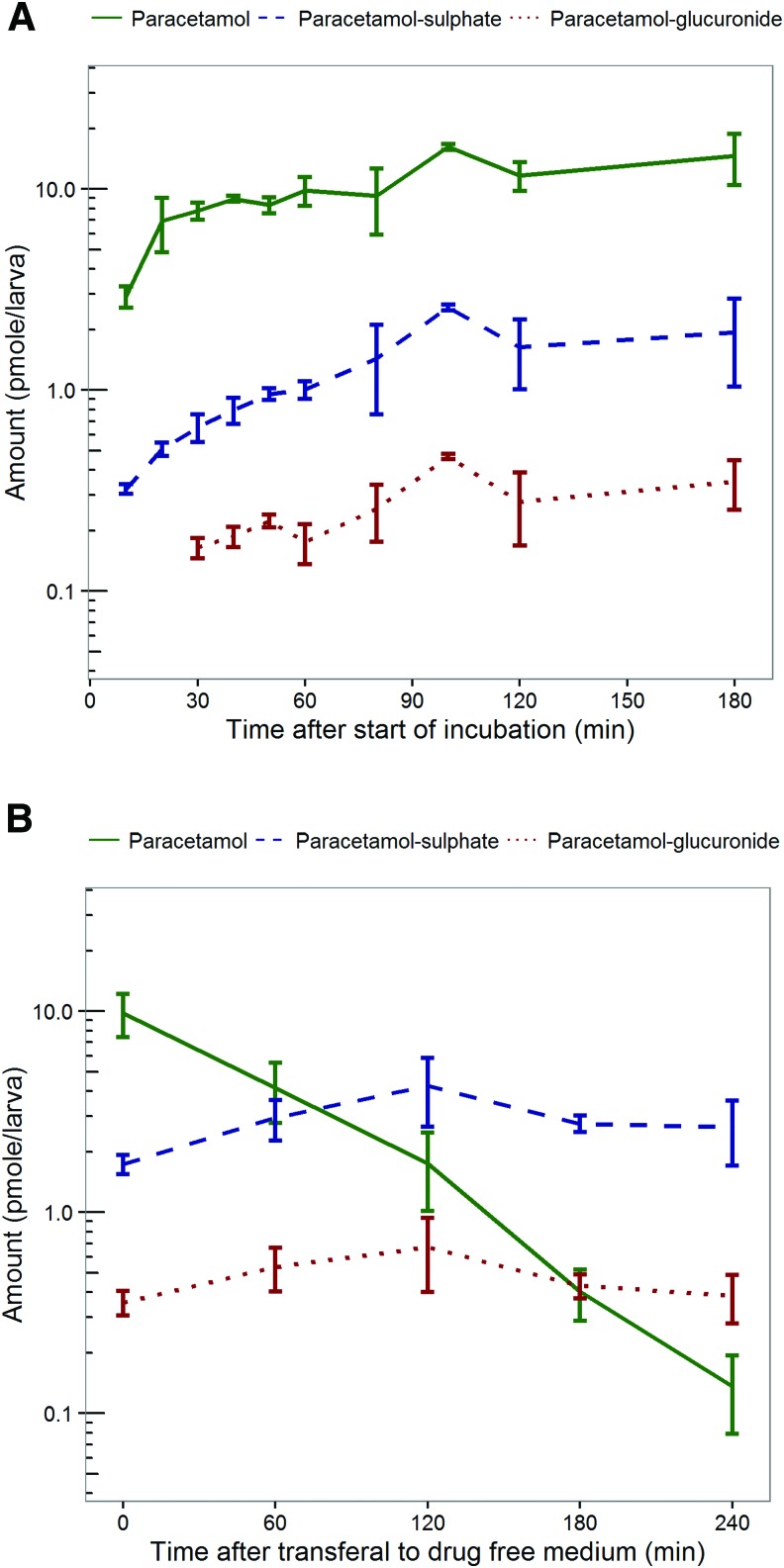
**(A**, **B)** Time course of amounts of paracetamol and its major metabolites in zebrafish larvae. Depicted are mean values ±1 standard deviations of paracetamol (*green solid line*), paracetamol-sulfate (*blue dashed line*), and paracetamol-glucuronide (*red dotted line*) amounts, after **(A)** continuous incubation in medium with 1 mM paracetamol (experiment 1), and **(B)** 1-h incubation in medium with 1 mM paracetamol and subsequent transferal to drug-free medium (experiment 2). Color images available online at www.liebertpub.com/zeb

Figure 1a shows the time course of the amount of paracetamol and its two major metabolites in the zebrafish larvae at 3 dpf, obtained after continuous exposure for various durations in medium with 1 mM paracetamol. The amount of paracetamol in the zebrafish larvae reached a plateau within 2 h, indicating that after 2 h the amount of paracetamol taken up by the larvae and the amounts metabolized and excreted by the larvae are in equilibrium. Paracetamol-sulfate and paracetamol-glucuronide amounts in zebrafish larvae could be detected after 10 and 30 min, respectively. These metabolites reached equilibrium between formation and excretion within 2 h as well.

Figure 1b describes amounts of paracetamol and its major metabolites in the zebrafish larvae at 3 dpf following transfer to drug-free medium after 1-h incubation in drug-containing medium. After transfer to drug-free medium, paracetamol amounts in the zebrafish larvae decreased over time. The amounts of the paracetamol metabolites in the larvae increased initially, indicating that per time unit larger amounts are formed than excreted. After 2 h in drug-free medium the elimination of the metabolites started to exceed the formation, resulting in peak amounts followed by a decline.

In both experiments, the amount of excreted paracetamol-glucuronide in the incubation medium was below the lower limit of quantification (0.5 pmole excreted per larva) throughout the experiment. In the first experiment, excreted sulfate metabolite was detected after 3 h of continuous exposure, but the level remained close to the limit of quantification (0.4 pmole excreted per larva). For the second experiment, excreted amounts of sulfate metabolite that were close to the limit of quantification were detected in the medium 3 h after the larvae were transferred from the drug-containing medium, to the drug-free medium. The excreted amounts are relatively low compared to the total amount of paracetamol and metabolites measured in the larvae, from which it can be concluded that a significant portion of the elimination of paracetamol is covered by measuring the sulfate and glucuronide metabolite in the larvae. Moreover, control experiments confirmed that paracetamol does not adhere to the skin of the zebrafish larvae, ratifying that paracetamol amounts quantified in the zebrafish larvae samples at 3 dpf indeed represent drug amounts taken up by the larvae.

### Nonlinear mixed effects model

The time course of paracetamol amounts in zebrafish larvae at 3 dpf was best described by a standard one-compartment distribution model, with 0-order absorption, and linear first-order elimination. It was found that the zebrafish larvae in a medium with a 1 mM paracetamol concentration take up 0.289 pmole paracetamol per minute. Every minute, the amount of paracetamol in 1.70% of the volume of the zebrafish larvae was cleared; this includes both metabolism and excretion of the unchanged drug. The obtained parameter values including the relative standard error of these estimates are provided in Table 1.

**Table 1.** Pharmacokinetic Model Parameter Estimates Obtained with the Population Pharmacokinetic Model

*Parameter*	*Unit*	*Parameter estimate*	*Relative standard error of estimate (%)*
Absorption rate constant (*k*_a_)	pmole/min	0.289	5.0
Distribution volume (*V*_1_)	Larva	1 FIX	—
Clearance (CL)	Proportion of larva volume/min	0.017	5.0
Residual error (variance)		9.73	19

**FIG. 2.**
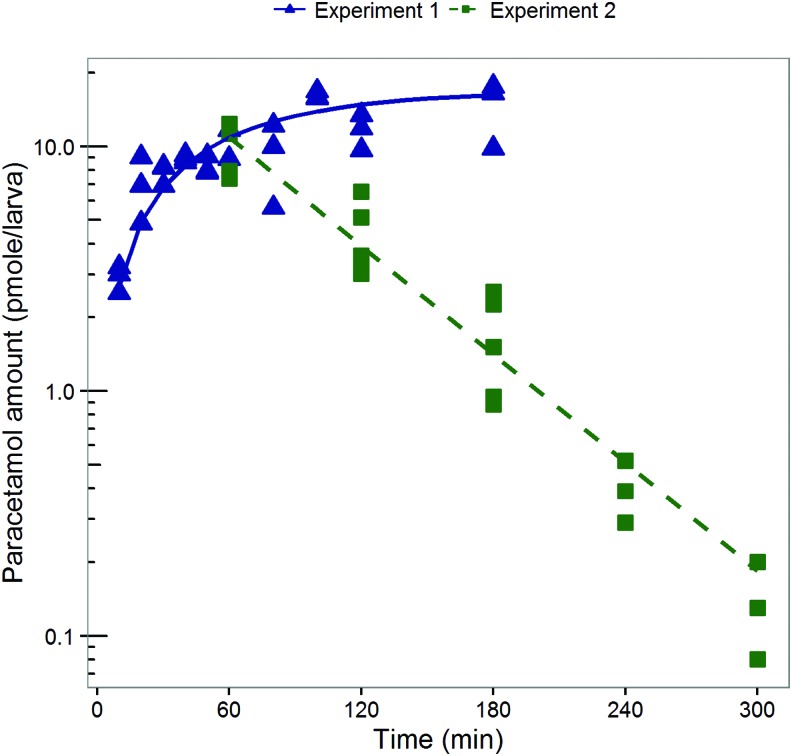
Observed and model predicted paracetamol amounts in zebrafish larvae. Observed (*symbols*) and model predicted (*lines*) paracetamol amounts in zebrafish larvae over time after continuous incubation in medium with 1 mM paracetamol (experiment 1, *blue solid line* and *triangles*) and after 1 h incubation in medium with 1 mM paracetamol and subsequent transfer to drug-free medium (experiment 2, *green dotted line* and *squares*). Color images available online at www.liebertpub.com/zeb

The relative standard errors in the obtained model parameter estimates were below 20%, indicating good precision of the parameter estimates. Figure 2 shows how paracetamol amounts change over time in experiments 1 and 2 according to the obtained nonlinear mixed effects PK model, and the amounts that were observed during the experiments. Goodness-of-fit plots are provided in the Supplementary Figure S1 (Supplementary Data are available online at www.liebertpub.com/zeb).

### Paracetamol clearance in zebrafish larvae

The average wet weight of the zebrafish larvae at 3 dpf was found to be 0.291 mg, and from this an average volume of 0.290 μL was derived. This yields absolute paracetamol clearance in zebrafish larvae at 3 dpf of 2.96 × 10^7^ L/h.

### Comparing paracetamol clearance in vertebrate species

**FIG. 3.**
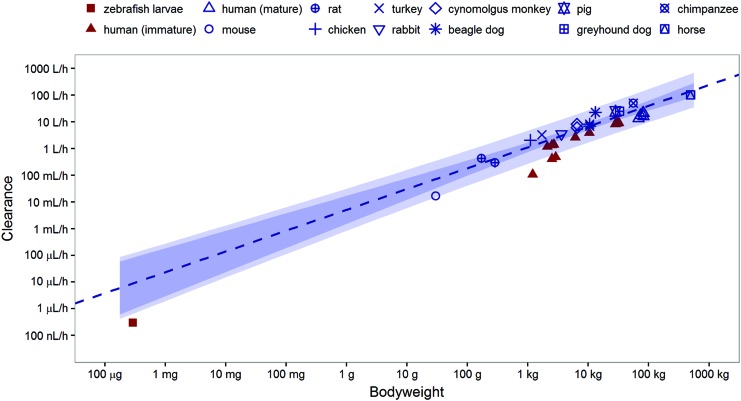
Paracetamol clearance across vertebrate species. Reported paracetamol clearances (*points*) and estimated allometric relationship (*dashed line*) between paracetamol clearance and bodyweight for mature individuals of various vertebrate species, including its 95% confidence (*darker shaded*) and prediction (*lighter shaded*) interval. Data from mature individuals of species are depicted in *blue*, from immature including the zebrafish larvae in *red*. Color images available online at www.liebertpub.com/zeb

Paracetamol CL reported over the past 10 year in vertebrate species, including humans of different ages, were obtained from literature. Supplementary Table S1, provides an overview of the obtained values. Figure 3 show a plot of these clearance versus the BW of each species.

The allometric equation that was fit to the data of mature individuals of the different species had an estimated exponent of 0.781 (*r*^2^ = 0.902). The obtained relationship is depicted in Figure 3 as well, including 95% confidence and prediction intervals and shows an over-prediction of the paracetamol clearance of the zebrafish larvae of 3 dpf. Interestingly, however, this relationship also over-predicts the reported paracetamol clearances in human newborns and children. When including both the mature and the immature individuals of all species except the zebrafish larvae, the exponent was estimated to be 0.838 (*r*^2^ = 0.835). Supplementary Figure S2 depicts this relationship and shows that the CL for paracetamol in zebrafish larvae obtained in this study does fall within the 95% confidence and prediction interval of this fit.

## Discussion

Analytical methods that can accurately measure concentrations of paracetamol and its metabolites in low volumes are a prerequisite for characterizing the PK of this drug in zebrafish larvae. We have developed an analytical method based on UPLC followed by mass spectrometric detection to separate and reliably quantify paracetamol and its major metabolite concentrations in samples of lysed zebrafish larvae at 3 dpf. The data obtained with this technique enabled the quantification of PK processes in this species with a nonlinear mixed effects modeling approach, proving for the first time the feasibility of studies on drug PK and metabolism in zebrafish larvae.

Quantification of PK processes in zebrafish larvae will be invaluable for drug screening procedures in drug discovery and early drug development. This is because it has been recognized in higher species and humans that drug concentrations rather than drug doses are driving drug effects, screening studies in zebrafish larvae generally ignore drug PK, or if these aspects are considered, drug concentrations are measured at a single time point, ignoring the full time course of drug and/or metabolite exposure.^3,17^ Combining information on full PK profiles with information on drug effects, is absolutely essential for the establishment of concentration–effect relationships, which are needed in translational pharmacology to scale drug effects between (vertebrate) species. Given that 70% of human genes have at least one obvious zebrafish ortholog^18^ the potential for a strong resemblance between humans and zebrafish larvae regarding disease manifestation and drug response is high. The zebrafish larvae has, for instance, already been proven useful in studying drug effects in diseases like tuberculosis^17^ and cancer.^2^

In addition to improving the translation potential of drug effects from zebrafish larvae to higher vertebrate species, it was investigated to what extent PK studies in zebrafish larvae could serve as a convenient translational platform for studies on drug metabolism as well. The comparison of our findings on paracetamol clearance in zebrafish larvae at 3 dpf and reported CL in higher vertebrates in Figure 3, shows that the clearance of paracetamol in the zebrafish larvae is lower than can be expected based on CL obtained in mature individuals of higher vertebrates, but that this value correlates well to values obtained in higher vertebrates when also values of immature individual are considered.

Zebrafish expresses phase II metabolizing genes similar to mammalian uridine glucuronosyltransferases and sulfotransferases isoforms throughout early development.^19–21^ Previous studies using different probes in zebrafish larvae, also suggest quantitative similarities in metabolite formation between zebrafish larvae and humans.^22–24^ Our quantitative results on the metabolism of paracetamol in zebrafish larvae at 3 dpf do, however, not closely resemble observed metabolic profiles in adult humans. As the graphs in Figure 1 show, paracetamol-sulfate concentrations are about five to six times higher than the concentrations of the glucuronide metabolite, which is contrary to findings in humans, in which the glucuronide is the predominantly formed metabolite for paracetamol. This could possibly be attributed to the immaturity of the enzymes in zebrafish larvae at 3 dpf. In human newborns, glucuronidation capacity is, for instance, known to be limited, resulting in an increased relative contribution of sulfation in the metabolism of paracetamol, causing the sulfate metabolite to be formed 3.5–8 times more than the glucuronide metabolites in this immature human population.^25–27^ Our experiments in the zebrafish larvae of 3 dpf were also performed before maturity in this species is reached. Although the dissimilarities in metabolic profiles between human adults and zebrafish larvae do not limit the applicability of our developed methodology in characterizing drug PK and determining accurate drug exposure in these larvae, it would be of interest to investigate if older larvae resemble metabolic profiles of human adults more closely. This is part of future investigations, and investigations on the clearance rates of other compounds in the zebrafish larvae.

In conclusion, we have successfully developed a feasible methodology for PK studies in zebrafish larvae at 3 dpf. The quantification of PK processes in these larvae by using a combination of ultra-sensitive LC-MS/MS-based analytical methods and nonlinear mixed effects modeling will allow the extraction of more information from experiments in zebrafish larvae during drug discovery and early drug development, which may yield advantages regarding the inter-species translation potential of findings on drug effects in these experiments.

## Supplementary Material

Supplemental data
