# Supplementary material for: Pharmacokinetic Modeling of Paracetamol Uptake and Clearance in Zebrafish Larvae: Expanding the Allometric Scale in Vertebrates with Five Orders of Magnitude
Source: Zebrafish. 2016 Dec 1;13(6):504–10. doi: 10.1089/zeb.2016.1313 (PMC5124745; doi:10.1089/zeb.2016.1313)
Supplement: Supplemental data [file Supp_Data.pdf]

Supplementary Data

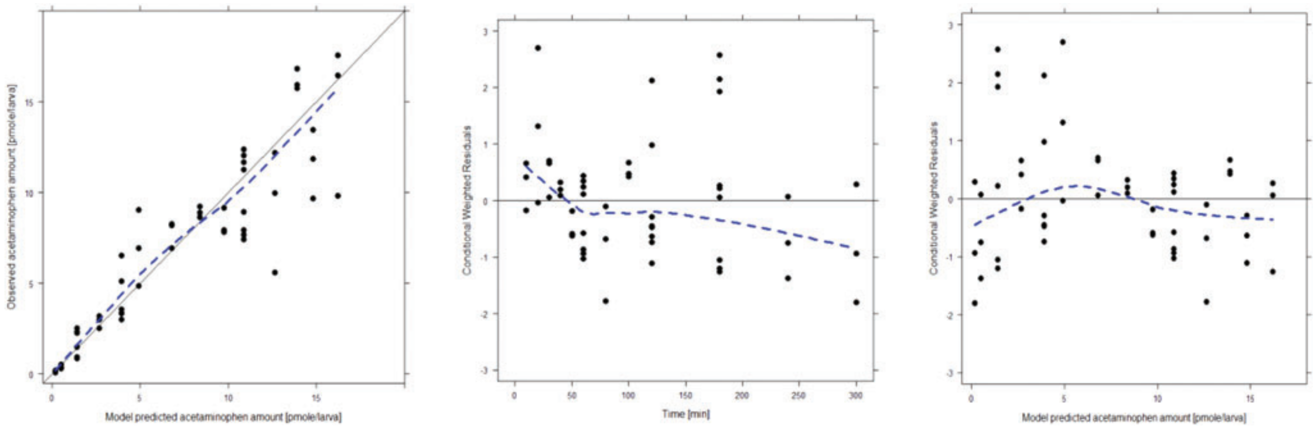

SUPPLEMENTARY FIG. S1. Basic goodness-of-fit plots with loess curves. *Blue dotted lines* for the population pharmacokinetic model of acetaminophen in zebrafish larvae, including observed versus predicted acetaminophen amounts (*left*), conditional weighted residuals versus time (*middle*), and conditional weighted residuals versus predicted concentration (*right*).

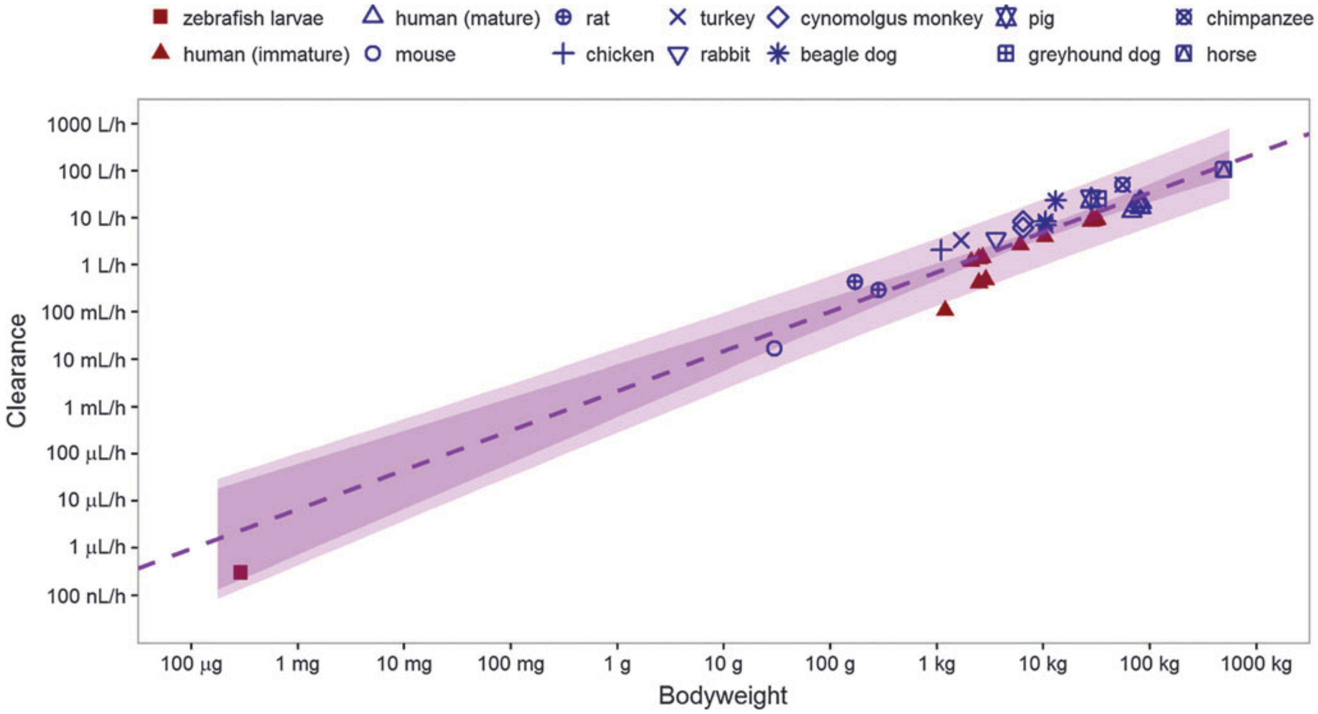

SUPPLEMENTARY FIG. S2. Paracetamol clearance across mature and immature vertebrate species Reported paracetamol clearances (*points*) and estimated allometric relationship (*dashed line*) between paracetamol clearance and bodyweight for both mature and immature individuals of various vertebrate species, including its 95% confidence (*darker shaded*) and prediction (*lighter shaded*) interval. Data from mature individuals of species are depicted in *blue*, from immature including the zebrafish larvae in *red*.

Supplementary Table S1. Overview of Acetaminophen Clearance Values Reported Over the Past 10 Years in Various Higher Vertebrates

Species	*Weight (kg)*	*Reported CL (unit)*	*CL (L/h)*	*Reference*
Mouse	0.030	0.28 (mL/min)	0.0168	1,2
Rat	0.17	2.53 (L/h/kg)	0.430	2,3
Rat	0.285	1.03 (L/h/kg)	0.294	4
Chicken	1.1	1.89 (L/h/kg)	2.08	5
Turkey	1.7	1.95 (L/h/kg)	3.32	5
Rabbit	3.61	3.52 (L/h)	3.52	6
Cynomolgus monkey	6.5	21.1 (mL/min/kg)	8.23	7,8
Cynomolgus monkey	6.5	15.7 (mL/min/kg)	6.12	7,8
Beagle dog	13.1	1.74 (L/h/kg)	22.8	5
Beagle dog	10.5	13.2 (mL/min/kg)	8.32	7
Beagle dog	10.5	11.1 (mL/min/kg)	6.99	7
Pig	28.1	0.88 (L/h/kg)	24.7	5
Greyhound dog	33	12.7 (mL/min/kg)	25.0	9
Greyhound dog	34	17.7 (mL/min/kg)	36.1	10
Chimpanzee	55.6	0.91 (L/h/kg)	50.6	11
Human (newborn)	1.2	0.0894 (L/h/kg)	0.107	12
Human (newborn)	2.1	16.3 (L/h/70 $kg^{0.75}$)	1.17	13
Human (newborn)	2.3	0.348 (L/h)	0.348	14
Human (newborn)	2.5	5 (L/h/70 $kg^{0.75}$)	0.411	15
Human (newborn)	2.5	16.3 (L/h/70 $kg^{0.75}$)	1.34	13
Human (newborn)	2.7	16.3 (L/h/70 $kg^{0.75}$)	1.42	13
Human (newborn)	2.9	5.24 (L/h/70 $kg^{0.75}$)	0.481	16
Human (child)	6.1	16.3 (L/h/70 $kg^{0.75}$)	2.61	13
Human (child)	10.4	16.3 (L/h/70 $kg^{0.75}$)	3.90	13
Human (child)	27.9	16.3 (L/h/70 $kg^{0.75}$)	8.18	13
Human (child)	29.3	16.5 (L/h/70 $kg^{0.75}$)	8.59	17
Human (child)	30.9	16.3 (L/h/70 $kg^{0.75}$)	8.83	13
Human (child)	32.3	16.3 (L/h/70 $kg^{0.75}$)	9.13	13
Human (adult)	68	3.3 (mL/min/kg)	13.5	18
Human (adult)	73	17.4 (L/h/70 $kg^{0.75}$)	18.0	19
Human (adult)	81	4.6 (mL/min/kg)	22.4	18
Human (adult)	83	4.2 (mL/min/kg)	20.9	18
Human (adult)	83	3.3 (mL/min/kg)	16.2	18
Horse	495	0.21 (L/h/kg)	104	5

CL, clearance values.

References

1. Saini SPS, Zhang B, Niu Y, Jiang M, Gao J, Zhai Y, *et al.* Activation of liver X receptor increases acetaminophen clearance and prevents its toxicity in mice. Hepatology 2011;54:2208–2217.
2. Van Zutphen LFM, Baumans VBA: Handboek proefdierkunde, 2rd ed. Elsevier gezondheidszorg, Maarssen, 2001.
3. Yamasaki I, Uotsu N, Yamaguchi K, Takayanagi R, Yamada Y. Effects of kale ingestion on pharmacokinetics of acetaminophen in rats. Biomed Res 2011;32:357–362.
4. Lee SH, An JH, Lee HJ, Jung BH. Evaluation of pharmacokinetic differences of acetaminophen in pseudo germ-free rats. Biopharm Drug Dispos 2012;33:292–303.
5. Neirinckx E, Vervaet C, De Boever S, Remon JP, Gommeren K, Daminet S, *et al.* Species comparison of oral bioavailability, first-pass metabolism and pharmacokinetics of acetaminophen. Res Vet Sci 2010;89:113–119.
6. Bienert A, Kamińska A, Olszewski J, Gracz J, Grabowski T, Wolc A, *et al.* Pharmacokinetics and ocular disposition of paracetamol and paracetamol glucuronide in rabbits with diabetes mellitus induced by alloxan. Pharmacol Rep 2012;64:421–427.
7. Koyanagi T, Yamaura Y, Yano K, Kim S, Yamazaki H. Age-related pharmacokinetic changes of acetaminophen, antipyrine, diazepam, diphenhydramine, and ofloxacin in male cynomolgus monkeys and beagle dogs. Xenobiotica 2014;44:893–901.
8. Cawthon Lang K: Primate Factsheets: Long-Tailed Macaque (Macaca fascicularis) Taxonomy, Morphology, & Ecology, 2006.
9. KuKanich B. Pharmacokinetics of acetaminophen, codeine, and the codeine metabolites morphine and codeine-6-glucuronide in healthy Greyhound dogs. J Vet Pharmacol Ther 2010;33:15–21.
10. KuKanich B. Pharmacokinetics and pharmacodynamics of oral acetaminophen in combination with codeine in healthy Greyhound dogs. J Vet Pharmacol Ther 2016;39:514–517.
11. Wong H, Grace JE, Wright MR, Browning MR, Grossman SJ, Bai SA, *et al.* Glucuronidation in the chimpanzee (Pan troglodytes): studies with acetaminophen, oestradiol and morphine. Xenobiotica 2006;36:1178–1190.
12. van Ganzewinkel C, Derijks L, Anand KJS, van Lingen RA, Neef C, Kramer BW, *et al.* Multiple intravenous doses of paracetamol result in a predictable pharmacokinetic profile in very preterm infants. Acta Paediatr 2014;103: 612–617.

13. Anderson BJ, Pons G, Autret-Leca E, Allegaert K, Boccard E. Pediatric intravenous paracetamol (propacetamol) pharmacokinetics: a population analysis. Paediatr Anaesth 2005; 15:282–292.
14. Cook SF, Roberts JK, Samiee-Zafarghandy S, Stockmann C, King AD, Deutsch N, *et al.* Population pharmacokinetics of intravenous paracetamol (acetaminophen) in preterm and term neonates: model development and external evaluation. Clin Pharmacokinet 2016;55:107–119.
15. Allegaert K, Palmer GM, Anderson BJ. The pharmacokinetics of intravenous paracetamol in neonates: size matters most. Arch Dis Child 2011;96:575–580.
16. Palmer GM, Atkins M, Anderson BJ, Smith KR, Culnane TJ, McNally CM, *et al.* I.V. acetaminophen pharmacokinetics in neonates after multiple doses. Br J Anaesth 2008; 101:523–530.
17. Mohammed BS, Engelhardt T, Cameron GA, Cameron L, Hawksworth GM, Hawwa AF, *et al.* Population pharmacokinetics of single-dose intravenous paracetamol in children. Br J Anaesth 2012;108:823–829.
18. Liukas A, Kuusniemi K, Aantaa R, Virolainen P, Niemi M, Neuvonen PJ, *et al.* Pharmacokinetics of intravenous paracetamol in elderly patients. Clin Pharmacokinet 2011;50: 121–129.
19. Allegaert K, Olkkola KT, Owens KH, Van de Velde M, de Maat MM, Anderson BJ, *et al.* Covariates of intravenous paracetamol pharmacokinetics in adults. BMC Anesthesiol 2014;14:77.
